# Retraction: Olefin epoxidation with chiral salen Mn(iii) immobilized on ZnPS-PVPA upon alkyldiamine

**DOI:** 10.1039/d0ra90126a

**Published:** 2020-11-26

**Authors:** J. Huang, D. W. Qi, J. L. Cai, X. H. Chen

**Affiliations:** Research Center for Advanced Computation, College of Science, Xihua University Chengdu 610039 PR China hj41012@163.com; Institute for Clean Energy & Advanced Materials, Southwest University Chongqing 400715 PR China; College of Rongchang, Southwest University Chongqing 402460 PR China; Ba Shu Middle School Chongqing 400023 PR China

## Abstract

Retraction of ‘Olefin epoxidation with chiral salen Mn(iii) immobilized on ZnPS-PVPA upon alkyldiamine’ by J. Huang *et al.*, *RSC Adv.*, 2016, **6**, 19507–19514, DOI: 10.1039/C6RA00002A.

The Royal Society of Chemistry, with the agreement of the authors, hereby wholly retracts this *RSC Advances* article due to extensive overlap with other published articles by these authors, including the text, data and figures published in ref. 1, which was not cited in this article. Although there are sections of original work, there are significant portions of text overlap, particularly in the Results and discussion section. Fig. 1, 3, 4 and 5, Tables 1 and 2 and Schemes 1 and 2 in the *RSC Advances* article have also been reproduced from ref. 1.

Signed: J. Huang, D. W. Qi, J. L. Cai and X. H. Chen

Date: 19^th^ November 2020

Retraction endorsed by Laura Fisher, Executive Editor, *RSC Advances*

## Supplementary Material
